# Syringic Acid Alleviates Doxorubicin-Induced Hepatotoxicity Through PI3K/Akt-Mediated Nrf-2/HO-1 Signaling Pathways in Male Rats

**DOI:** 10.3390/ijms26167779

**Published:** 2025-08-12

**Authors:** Maha Abdullah Alwaili, Thamir M. Eid, Amal S. Abu-Almakarem, Alaa Muqbil Alsirhani, Noorah Saleh Al-Sowayan, Rabab Mohamed Aljarari, Effat A. Al-Judaibi, Aljazi Abdullah AlRashidi, Maysa A. Mobasher, Karim Samy El-Said

**Affiliations:** 1 Department of Biology, College of Science, Princess Nourah bint Abdulrahman University, Riyadh 11671, Saudi Arabia; maalwaele@pnu.edu.sa; 2 Department of Biochemistry, Faculty of Science, King Abdulaziz University, Jeddah 21589, Saudi Arabia; tmeid@kau.edu.sa; 3 Experimental Biochemistry Unit, King Fahad Medical Research Center, King Abdulaziz University, Jeddah 21589, Saudi Arabia; 4 Department of Basic Medical Sciences, Faculty of Applied Medical Sciences, Al-Baha University, Al-Baha 65525, Saudi Arabia; amala2050@yahoo.com; 5 Department of Chemistry, College of Science, Jouf University, Sakaka 72341, Saudi Arabia; amassaf@ju.edu.sa; 6 Department of Biology, College of Science, Qassim University, Buraidah 52377, Saudi Arabia; 7 Department of Biological Sciences, College of Science, University of Jeddah, Jeddah 21959, Saudi Arabia; rmaljerary@uj.edu.sa (R.M.A.); eaaljedeibi@uj.edu.sa (E.A.A.-J.); 8 Chemistry Department, Faculty of Science, University of Ha’il, Ha’il 81451, Saudi Arabia; a.alrashedy@uoh.edu.sa; 9 Department of Pathology, Biochemistry Division, College of Medicine, Jouf University, Sakaka 72388, Saudi Arabia; mmobasher@ju.edu.sa; 10 Biochemistry Division, Chemistry Department, Faculty of Science, Tanta University, Tanta 31527, Egypt

**Keywords:** syringic acid, antioxidants, anti-inflammatories, doxorubicin, hepatotoxicity

## Abstract

Syringic acid (SYA) is a significant phenolic compound with the potential for various biomedical uses, including uses of its hepatoprotective properties. Doxorubicin (DOX) is a drug used in the treatment of several tumors, but its side effects, particularly hepatotoxicity, limit its effectiveness. This study investigated the therapeutic effects of SYA on DOX-induced hepatic injury in rats. Molecular docking studies were performed using AutoDock Vina. Five groups of Sprague–Dawley rats (eight in each group) were studied. Gp1 was a negative control group; Gps2–5 was administered intraperitoneally (i.p.) with DOX at a dosage of 4 mg/kg once a week for a month; and Gp2 was left as a positive control group. Gps3–5 received oral SYA at doses of 25, 50, or 75 mg/kg/day, respectively, for a month. Histopathological, molecular, and biochemical analyses were conducted one month after the last SYA dosages were given. The findings demonstrated that by reversing biochemical changes and reducing oxidative stress and inflammation, SYA therapy considerably reduced DOX-induced hepatotoxicity in rats. These results implied that SYA may lessen the hepatotoxicity that DOX causes in rats.

## 1. Introduction

Doxorubicin (DOX) is one of the most used chemotherapies for treating various malignancies due to its multiple mechanisms [1]. This chemotherapeutic antibiotic, a member of the anthracycline class, causes toxicity by killing both cancerous and non-cancerous cells, even in organs not targeted. The hepatotoxic effects of this drug are complex and limit its effectiveness; however, they can result in the accumulation of hazardous intermediates and the development of oxidative stress and inflammation. Changes in hepatic tissues treated with DOX include hepatocyte vacuolation and degeneration and bile duct necrosis [2]. DOX worsens oxidative stress by inhibiting transcription factors and modulating signaling pathways that regulate cellular homeostasis [3]. Various signaling pathways, including the PI3K/Akt pathway, modulate hepatocytic oxidative stress by targeting GSK-3β, and this suggests that they may have promise in combating DOX-induced hepatotoxicity [4]. DOX has a well-established reputation as a hepatotoxic agent in both preclinical and clinical contexts, and formal RUCAM-based assessments of drug-induced liver injury (DILI) due to DOX have also been reported. In many clinical cases, multiple agents, including DOX, have been co-administered, complicating situations and curtailing the ability of researchers to determine which single drug is causing the liver injury. In a case series reported by Danan and Teschke (2016), RUCAM scoring was applied retrospectively to patients receiving DOX. DOX was rated as a “possible” or “probable” contributor to hepatotoxicity based on the timing of administration and the exclusion of other causes [5,6]. Herbal-derived substances have been explored and shown to be effective as potential adjuvant treatments for preventing DOX-triggered hepatotoxicity because of their antioxidant and anti-inflammatory properties [7]. The beneficial effects of berberine against DOX-promoted hepatotoxicity in mice have been reported [8]. Song et al. (2019) demonstrated the effects of dioscin on DOX-induced hepatotoxicity by controlling the Sirt1/FOXO1/NF-κ*B* signaling pathway [9]. A previous study indicated that citronellal alleviated DOX-triggered hepatotoxicity via antioxidative stress in rats [10]. Another report showed that rutin and quercetin could potentially have therapeutic effects against hepatic injury induced by DOX through their modulation of Nrf-2 expression in rats [11]. Furthermore, avenanthramide-C, a natural plant-derived compound, had an ameliorative effect against DOX-induced hepatotoxicity in rats by modulating the Akt/GSK-3β pathway [12].

The phenolic secondary metabolites widely produced by plants are phytochemicals that could potentially be used to control various disorders that have garnered considerable public and scientific interest, including liver disorders [13]. Of these phenolics, syringic acid (SYA) is one found in abundance in several plants. It has useful properties in pharmacological and biomedical applications, such as antioxidants, anti-inflammatory, cardioprotective, and hepatoprotective properties [14]. Mirza et al. (2019) reported that SYA (2000 mg/kg/p.o.) did not show any mortality or signs of toxic effects in rats [15]. Recently, it was shown that SYA’s anti-inflammatory and antioxidant actions are mediated through the Nrf-2 signaling pathway, and that this mediation facilitates the restoration of the endogenous antioxidant system in rats with hepato-testicular damage caused by methyl cellosolve [16,17]. SYA has been reported to alleviate hepato-renal injuries induced in experimental animals [18]. Additionally, SYA has been reported to possess hepatoprotective effects against thioacetamide-induced hepatic encephalopathy in rats [19]. SYA had potential hepatoprotective efficacy in rats with non-alcoholic fatty liver through its modulation of the Nrf-2/HO-1 signaling pathway [20]. Previous studies reported that among different dosages given, a dosage of SYA at 50 mg/kg significantly protected against hepatic damage in rats [20,21].

Interestingly, SYA demonstrated better antioxidative effects than ascorbic acid in dimethyl nitrosamine-induced hepatotoxicity in rats [22]. Additionally, Rashedinia et al. (2020) reported the regulation of metabolism by SYA, beyond its antioxidant role, in the brains and spinal tissues of diabetic rats [23]. The effect of SYA on oxidative stress and inflammatory pathways against lead acetate-triggered testicular injury has been reported in rats [24]. SYA inhibited cancer cell proliferation, inflammation, and apoptosis that had been induced by targeting the Akt/mTOR signaling pathway [25]. Liu et al. (2021) reported the chemoprotective effect of SYA on cyclophosphamide-induced ovarian damage that was due to the drug’s anti-inflammatory characteristics [26]. Furthermore, SYA demonstrated potential protection against cisplatin-induced nephrotoxicity in rats [27]. Given the need to develop a new strategy to reduce the hepatotoxicity induced by DOX, there is hope for the use of plant-based bioactive phytochemicals, which could be highly efficient for this purpose. Therefore, this study investigated for the first time the efficacy of SYA in ameliorating DOX-induced hepatic injury through its mitigation of oxidative stress and inflammation in male rats.

## 2. Results

### 2.1. In Silico ADMET Analysis for SYA and DOX

**Figure 1:**
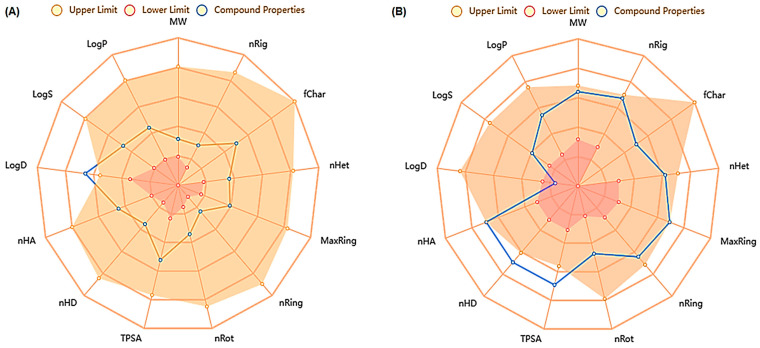
Radar chart for ADMET of SYA (**A**) and DOX (**B**).

In silico ADMET analysis further complemented the docking studies by providing crucial information about the pharmacokinetics and toxicity profiles of SYA and DOX. The results revealed that SYA exhibited favorable drug-likeness and safety properties. It had a good rating in terms of Lipinski’s Rule of Five, with a molecular weight of 198.05 g/mol, low lipophilicity (LogP = 1.21), and a moderate topological polar surface area (TPSA = 75.99 Å^2^), indicating good oral bioavailability. In contrast, DOX had a poor Rule-of-Five rating, primarily owing to its high molecular weight (543.17 g/mol), large TPSA (212.39 Å^2^), and elevated hydrogen bond counts, suggesting poor oral absorption. SYA demonstrated low P-glycoprotein substrate and inhibitor probabilities (0.003 and 0.002, respectively), which thereby predicted minimal risks of efflux and drug–drug interactions. Although both compounds had low percentages of human intestinal absorption (HIA for SYA = 0.028%; DOX = 0.751%), SYA had slightly better permeability in Caco-2 (−5.142) and MDCK assays (1.09 × 10^5^) compared with DOX. The plasma protein binding percentage (PPB%) of SYA was moderate (50.89%), whereas DOX showed a higher percentage of binding (91.29%). This may have influenced the free drug availability and toxicity of DOX. Predictions of the penetration of the blood–brain barrier (BBB) indicated limited central nervous system exposure for DOX (0.011), which could be advantageous for reducing off-target effects in non-CNS applications. Regarding the metabolism, SYA exhibited minimal inhibition or substrate affinity for major cytochrome P_450_ enzymes, including CYP3A4, CYP2D6, and CYP1A2, suggesting a low risk of metabolic interference. The cytochrome P_450_ interaction profiles revealed minimal inhibition potential across most isoforms for all compounds, suggesting a lower risk of drug–drug interactions. However, DOX showed moderate inhibition of CYP1A2 (0.489), and this finding warranted consideration in polypharmacy scenarios. On the other hand, DOX showed moderate interaction potential, especially as a substrate for multiple CYP isoforms. Interestingly, the clearance (CL) rate of SYA was 7.21 mL/min/kg, which was lower than that for DOX (9.57 mL/min/kg), indicating a relatively longer systemic retention. Toxicologically, SYA demonstrated a safer profile with a low predicted hepatotoxicity (DILI = 0.795), minimal cardiotoxicity (hERG inhibition = 0.034), and negligible mutagenicity (Ames = 0.009). In contrast, DOX showed significantly higher values for hepatotoxicity (DILI = 0.964), mutagenicity (Ames = 0.811), and carcinogenicity (0.776), in agreement with its known clinical toxicity. Notably, SYA had a high quantitative estimate of drug-likeness (QED = 0.76), whereas DOX scored much lower (QED = 0.147); this supported the favorable drug profile of SYA. According to these ADMET screenings, SYA rated well in terms of the Lipinski Rule of Five, but DOX did not. SYA’s ADMET qualities fell between the upper and lower expected values, according to the overall study, and this showed that SYA was more biodegradable and bioavailable than DOX (Figure 1 and Table 1).

### 2.2. Molecular Docking Interactions of SYA and DOX with PI3K, Akt, Nrf-2, and HO-1

**Figure 2:**
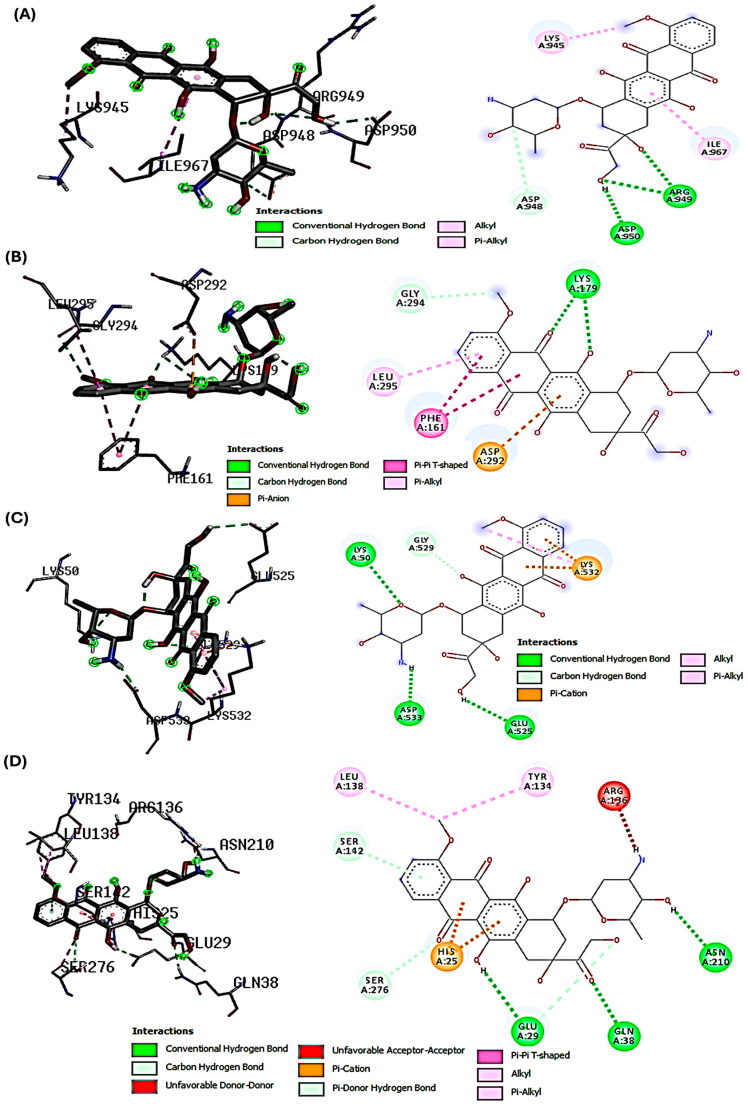
Interactions of DOX with PI3K, Akt, Nrf-2, and HO-1. (**A**) Interactions with PI3K—conventional H-bonds (ARG949 and ASP950); carbon H-bond (ASP948); and hydrophobic interactions (LYS945 and ILE967). (**B**) Interactions with Akt—conventional and carbon H-bonds (LYS179 and GLY294, respectively); electrostatic interaction (ASP292); and hydrophobic interactions (PHE161 and LEU295). (**C**) Interactions with Nrf-2—conventional H-bond (LYS50, GLU525, ARG60, and ASP533); carbon H-Bond (GLY529); and hydrophobic interactions (LYS532). (**D**) Interactions with HO-1—conventional H-bond (GLN38, GLU29, and ASN210); carbon H- Bond (GLU29 and SER276); and hydrophobic interactions (LYS532, LEU138, and TYR134).

**Figure 3:**
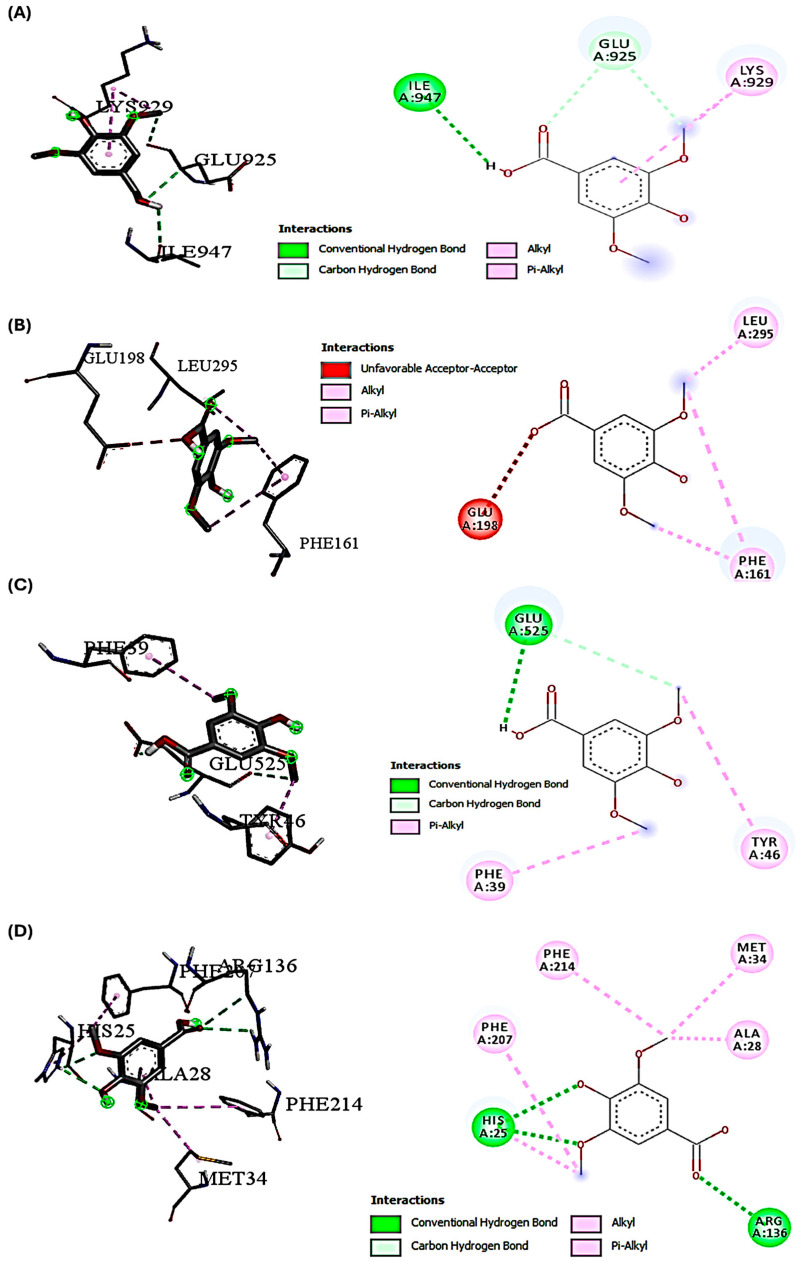
Interactions of SYA with PI3K, Akt, Nrf-2, and HO-1. (**A**) Interactions with PI3K—conventional and carbon H-bonds (ILE947 and GLU925) and hydrophobic interactions (LYS929). (**B**) Interactions with Akt—unfavorable acceptor–acceptor bonds (GLU198); and hydrophobic interactions (LEU925 and PHE161). (**C**) Interactions with Nrf-2—conventional H-bond (GLU525) and hydrophobic interactions (PHE39 and TYR46). (**D**) Interactions with HO-1—conventional H-bond (HIS25 and ARG136) and hydrophobic interactions (ALA28, MET34, PHE207, and PHE214).

The docking studies investigated the potential molecular interactions with key proteins involved in the PI3K/Akt-mediated Nrf-2/HO-1 signaling pathway. These interactions are relevant in the context of DOX-induced hepatotoxicity and the hepatoprotective effects of SYA on the modulation of signaling pathways affected by DOX-induced oxidative stress. Our primary aim was to demonstrate how SYA may interact with specific targets in this signaling cascade to activate antioxidant defense mechanisms that could counteract DOX-induced oxidative stress. The binding interactions between SYA and DOX with various protein targets were associated with important cellular pathways. Notably, DOX consistently demonstrated stronger binding affinities across all protein targets compared with SYA, with binding scores ranging from −6.5 to −7.9 kcal/mol (Table 2). This superior binding profile of DOX aligns with its established therapeutic efficacy and suggests potential molecular mechanisms underlying its biological activity. These interactions imply a potential mechanism for modulating the Nrf-2 pathway, which would be crucial for a cellular defense against oxidative stress. The molecular docking findings complemented our in vivo data and supported the proposed mechanism by which SYA activates cellular antioxidant response. The binding pattern observed with Akt (score −7.9 kcal/mol) provided additional mechanistic insights. The complex network of interactions, including conventional hydrogen bonds with LYS179 and pi-pi T-shaped interactions with PHE161, suggested the potential regulation of the PI3K/Akt signaling pathway. This interaction profile could explain how DOX and SYA might influence cellular pathways, actions that would be particularly relevant in therapeutic contexts. The structural analysis of protein-ligand interactions revealed a common theme of multiple hydrogen bonding networks across different protein targets. For instance, the HO-1 complex showed intricate interactions, including both conventional hydrogen bonds and pi-pi stacking, suggesting a stable binding mode that could influence the protein’s activity in the cellular stress response pathway. Similarly, the interactions observed with Nrf-2 demonstrated a complex network of hydrogen bonds and hydrophobic interactions that could modulate its transcriptional activity (Table 2 and Figure 2 and Figure 3).

### 2.3. Impact of DOX/SYA Therapy on Changes in Liver and Body Weight

The body weight change percentage (B.W.C%) of the animals challenged with DOX was significantly decreased (*p* < 0.05) by 13.94%, compared with that of the control group (38.71%). When compared with the rats exposed to DOX only, the rats injected with DOX and then treated with SYA at a dosage of 25, 50, or 75 mg/kg had significant improvements (*p* < 0.05) in the B.W.C% in a dose-dependent manner. The relative liver weight in the group given DOX was higher than in the study’s experimental groups (Table 3).

### 2.4. Treatment with SYA Has Mitigated Liver Dysfunction Induced by DOX in Rats

Table 4 indicates that the group injected with DOX had significantly higher levels of ALP and the liver transaminases (ALT and AST) (*p* < 0.05) than other experimental groups. It also showed that SYA therapy significantly decreased the levels of AST, ALT, and ALP in a dose-dependent manner. When compared to the control group (7.64 ± 0.79 U/L), the group exposed to DOX showed a significant increase (*p* < 0.05) in the level of GGT, at 19.14 ± 1.95 U/L. Treatment with SYA at 50 mg/kg resulted in a significant decrease in the serum GGT level (9.23 ± 1.17 U/L), which was close to normal levels when compared with the levels in the other treated groups. On the other hand, the total protein levels in the DOX-injected group dropped considerably (*p* < 0.05), but these levels recovered in the animals given DOX followed by SYA therapy (Table 4).

### 2.5. SYA Treatment Reduces DOX-Induced Hepatic Oxidative Stress in Rats

The hepatic MDA levels of rats injected with DOX (6.85 ± 0.57 nmol/mg protein) were significantly higher (*p* < 0.05) than those of the control group (2.16 ± 0.18 nmol/mg protein). On the other hand, the rats given DOX showed a significant decrease in the hepatic antioxidants: GSH, GST, GPX, and SOD. However, treatment with DOX/SYA effectively prevented (*p* < 0.05) an increase in liver lipid peroxidation (i.e., MDA levels), a decrease in the activities of SODs, GSTs, GPXs, and a decrease in the GSH levels in a concentration-dependent manner (Table 5).

### 2.6. Treatment with SYA Mitigates Inflammation in the Liver Tissues of DOX-Injected Rats

**Figure 4:**
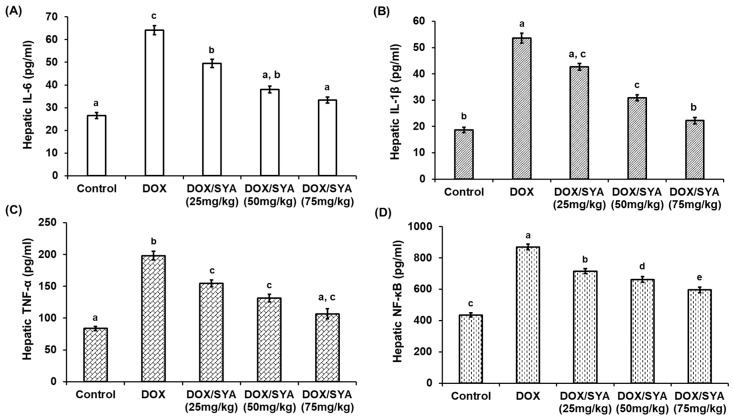
Hepatic interleukin-6 (IL-6) (**A**), interleukin-1β (IL-1β) (**B**), tumor necrosis factor-α (TNF-α) (**C**), and nuclear factor kappa-B (NF-κB) (**D**) in the different groups. DOX: Doxorubicin, SYA: Syringic acid. The values represent means ± SEM, (*n* = 8). Means with different lowercase letters indicate statistically significant differences between groups (*p* < 0.05), as determined by Tukey’s HSD post hoc test following one-way ANOVA. Groups sharing the same letter are not significantly different.

Levels of the inflammatory cytokines (IL-6, IL-1β, TNF-α, and NF-κB) were assessed in liver tissues after different treatments to explore the impact of SYA on DOX-induced inflammatory responses in rats’ livers. The findings demonstrated that, in comparison with the control group, the levels of these inflammatory biomarkers were considerably higher (*p* < 0.05). However, the group that received DOX/SYA showed a significant (*p* < 0.05) drop in these inflammatory cytokines, with the most notable improvement seen in the DOX/SYA group that received a SYA dosage of 75 mg/kg (Figure 4).

### 2.7. Treatment with SYA Targeting the PI3K/Akt-Mediated Nrf-2/HO-1 Pathway in DOX-Challenged Rats

**Figure 5:**
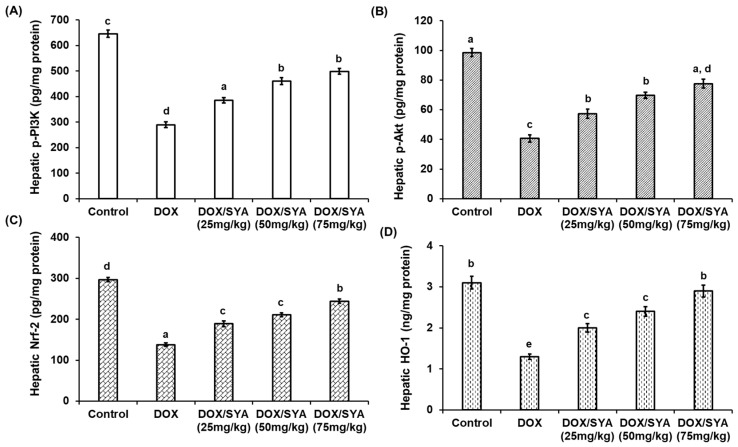
Hepatic levels of rats’ phosphorylated phosphoinositide 3-kinase (p-PI3K) (**A**), phosphorylated protein kinase B (p-Akt) (**B**), nuclear factor erythroid 2-related factor 2 (Nrf-2) (**C**), and heme oxygenase 1 (HO-1) (**D**) in the different groups. DOX: Doxorubicin, SYA: Syringic acid. The values represent means ± SEM (*n* = 8). Bars with different lowercase letters indicate statistically significant differences between groups (*p* < 0.05), as determined by Tukey’s HSD post hoc test following one-way ANOVA. Groups sharing the same letter are not significantly different.

**Figure 6:**
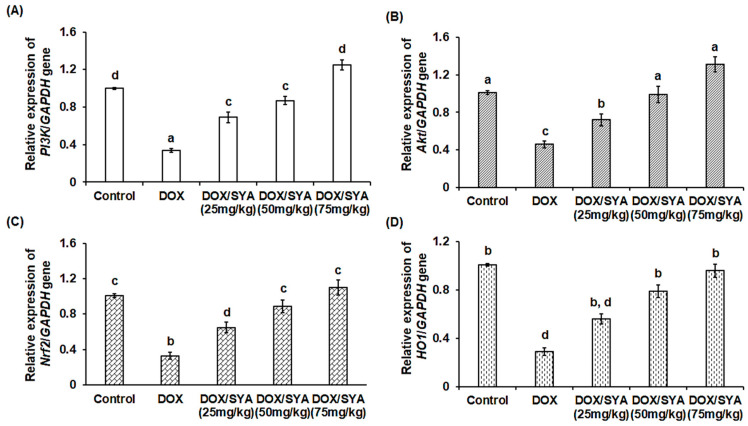
Relative mRNA expression levels of the hepatic phosphoinositide 3-kinase (*PI3K*) (**A**), RAC-alpha serine/threonine-protein kinase (*Akt*) (**B**), Nuclear factor erythroid 2-related factor 2 (*Nrf2*) (**C**), and Hem-oxygenase 1 (*HO1*) (**D**) in the different groups. DOX: Doxorubicin, SYA: Syringic acid. The values represent means ± SEM, (*n* = 8). Bars with different lowercase letters indicate statistically significant differences between groups (*p* < 0.01), as determined by Tukey’s HSD post hoc test following one-way ANOVA. Groups sharing the same letter are not significantly different.

The hepatic protein levels of phosphorylated PI3K and Akt were significantly decreased (*p* < 0.05) in the group injected with DOX to 289.67 ± 11.6 and 40.59 ± 2.58 pg/mg protein, respectively, compared with the negative control group (645.78  ±  14.3 and 98.59 pg/mg protein, respectively). Furthermore, the hepatic Nrf-2 and HO-1 protein levels were significantly decreased (*p* < 0.05) in the DOX-injected group (Nrf-2 at 138.23  ±  3.98 pg/mg protein and 1.31  ±  0.15 ng/mg protein, respectively) when compared to the normal control (297.19  ±  5.55 pg/mg protein and 3.12  ±  0.28 ng/mg protein, respectively). However, treatment with SYA led to significant increases in the p-PI3K, p-Akt, Nrf-2, and HO-1 protein levels in hepatic rats in a dose-dependent manner compared with the group injected only with DOX alone (Figure 5). Additionally, in terms of gene expression, the relative mRNA expressions of the *PI3K* and *Akt* genes were significantly downregulated (*p* < 0.01) in the rats injected with DOX by −2.9-fold or −2.2-fold, respectively, compared with the rats in the normal control group. Treatment with DOX and SYA led to a significant upregulation of these genes. Also, the *Nrf2* and *HO1* genes were significantly upregulated upon co-treatment with DOX and SYA, and the highest values were recorded when SYA was given at a dosage of 75 mg/kg (Figure 6).

### 2.8. Treatment with SYA Reduces Histopathological Alterations Caused by DOX in Rat Liver Tissues

**Figure 7:**
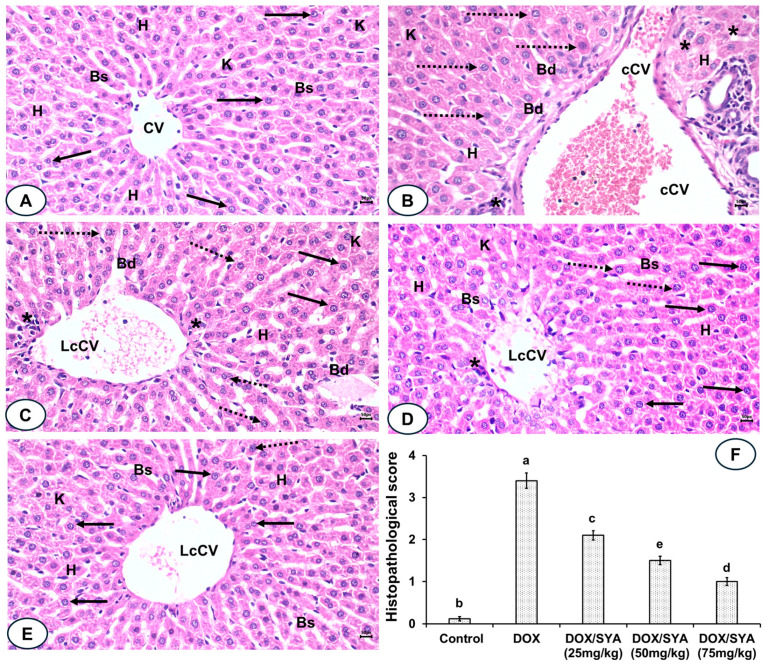
(**A**) A photomicrograph of the liver section of the normal control group shows normal-like structures of hepatic tissue with regular central veins (CV), centered nucleus (arrows), normal hepatocytes (H), blood sinusoids (Bs), and Kupffer cells (K). (**B**) The Liver section of the DOX-administered group displays distorted hepatic architecture, congested CV (cCV), pyknotic nuclei (dotted arrows), cellular infiltrations (*), blood sinusoids dilations (Bd), and distinct Kupffer cells (K). (**C**) Liver section of the DOX/SYA (25 mg/kg)-treated group exhibits some disorganization of hepatic architecture, less congested central veins (LcCV), some pyknotic nuclei (dotted arrows), and other centered nuclei (arrows). (**D**) Liver section of the DOX/SYA (50 mg/kg)-treated group shows improvement in the hepatic organization, less congested central veins (LcCV), and fewer binucleated hepatocytes (arrows). (**E**) Liver section of the DOX/SYA (75 mg/kg)-treated group shows improvement in the hepatic tissue’s structures, represented by less congestion in the central vein, and fewer hepatocytes containing pyknotic nuclei (dotted arrows), and fewer cellular infiltrations (H&E ×400, scale bar = 50 μm). (**F**) The histopathological scores of the different groups’ sections. The values represent means ± SEM, (*n* = 8). Bars with different lowercase letters indicate statistically significant differences between groups (*p* < 0.05), as determined by one-way ANOVA. Groups sharing the same letter are not significantly different.

Liver sections of the normal control group stained with H&E had normal-like hepatic structures, with a regular central hepatic vein, a centered nucleus, and normal blood sinusoids; the pathological scores were 0.10 ± 0.05 (Figure 7A,F). In contrast, the liver sections of DOX-injected rats exhibited extensive destruction of hepatocytes, along with a congested central vein, cellular swelling, dilated blood sinusoids, a pyknotic nucleus, and distinct Kupffer cells. A semi-quantitative analysis revealed a significant increase (*p* < 0.05) in their pathological score, which was recorded as a score of 3.40 ± 0.18 compared with the normal control group (see Figure 7B,F). The liver sections of the group given DOX and then SYA at 25 mg/kg displayed some disorganization of the hepatic architecture, less congested central veins (CV), some pyknotic nuclei, and other centered nuclei. A significant decrease (*p* < 0.05) in the pathological score (recorded as 2.1 ± 0.11) was observed compared with the sections from the rats given DOX (see Figure 7C,F). Similarly, the liver sections of the rats given DOX and then SYA at a dosage of 50 mg/kg showed a significant improvement in their hepatic structures and reduced levels of congestion, resulting in a lower pathological score (1.5 ± 0.10) compared with the group given DOX (see Figure 7D,F). Additionally, the liver sections of the group given DOX and then SYA at a dosage of 75 mg/kg exhibited a significant improvement in the hepatic histological alterations caused by DOX, with a notable decrease (*p* < 0.05) in the pathological score (recorded as 1.0 ± 0.09) (see Figure 7E,F).

## 3. Discussion

Although DOX is widely used and highly effective as an anticancer medication, it can cause serious damage to multiple organs. DOX–induced hepatotoxicity is classified as an intrinsic form of drug-induced liver injury (DILI). This classification is supported by its dose-dependent hepatotoxic effects, the reproducibility of its effects in animal models, and its well-characterized mechanisms leading to oxidative stress, mitochondrial dysfunction, and apoptosis. In both clinical and preclinical settings, hepatic injury caused by DOX is closely linked to the cumulative dose and treatment duration, consistent with the profile of intrinsic DILI [28]. DOX causes direct hepatocellular injury via the generation of reactive oxygen species (ROS), leading to lipid peroxidation, mitochondrial DNA damage, and impaired antioxidant defense systems; this finding supports the view that DOX hepatotoxicity is intrinsic [29]. The mechanistic basis of DOX-induced hepatotoxicity in humans involves several interrelated pathways, including oxidative stress and ROS generation, mitochondrial dysfunctions, inflammatory signaling activation, impaired antioxidant defenses, cholestasis, and impaired bile flow [30]. Therefore, it is crucial to minimize the negative effects of DOX (and all chemotherapeutic drugs) while maximizing its therapeutic benefits. Research has investigated the use of several medicines in combination with DOX to mitigate its adverse effects [12,31]. There are currently no effective therapeutic options for the hepatotoxicity associated with DOX. Natural herbal compounds may be valuable, unique pharmaceutical compounds for treating severe conditions [32]. Finding natural components that can enhance DOX’s therapeutic effectiveness while reducing its negative impact on healthy tissues is therefore essential. Plant-derived polyphenolic compounds, including SYA, have shown promising hepatoprotective effects and other pharmaceutical effects [33]. This study investigated the effectiveness of SYA in reducing the inflammation and oxidative stress caused by DOX-induced hepatic damage in rats. The data points toward idiosyncratic drug-induced autoimmune hepatitis (DIAIH) as the type of human DILI observed. A previous study delves into the DIAIH case series, emphasizing bimodal diagnostic criteria, such as RUCAM for assessing causality from drugs and autoimmune characteristics, which are hallmark features of drug-induced autoimmune hepatitis [34].

Understanding a molecule’s chemical reactivity characteristics is crucial for creating novel therapeutic medications [35]. In silico pharmacokinetics and ADMET studies were conducted to computationally determine the bioactivity properties of SYA and DOX. The results showed that SYA followed the Lipinski rules, but DOX did not. The analysis revealed that SYA was more biodegradable and bioavailable, consistent with the findings of previous ADMET screening studies [12,31]. In contrast, the in-silico predictions using ADMET supported the high DILI potential of DOX, consistent with its known intrinsic hepatotoxicity observed in both preclinical and clinical contexts. These ADMET findings reinforced the experimental results, highlighting SYA’s low toxicity, metabolic stability, and favorable pharmacokinetics, all of which may contribute to its protective effect against DOX-induced hepatotoxicity observed in vivo via activation of the PI3K/Akt-Nrf2/HO-1 pathway. A molecular docking analysis was conducted to investigate the potential molecular interactions between SYA and the key signaling proteins—PI3K, Akt, Nrf-2, and HO-1, which are central to the PI3K/Akt-mediated Nrf-2/HO-1 pathway, which regulates cellular antioxidant defenses. Docking interaction profiling explained how DOX and SYA might affect cellular pathways, particularly by targeting the PI3K, Akt, Nrf-2, and HO-1 proteins in a therapeutic context. A structural analysis of protein-ligand interactions showed multiple hydrogen bonding networks across different protein targets. Given that DOX is known to induce oxidative stress and hepatotoxicity through disruption of this pathway, it was included in the analysis as a reference compound. DOX formed a stable binding posture through hydrophobic and electrostatic interactions. Strong connections between DOX and the stress response and levels of antioxidant proteins may increase its potential for hepatotoxicity, as well as contribute to its therapeutic effects [31]. The docking results aimed to provide molecular insight into how SYA may modulate these signaling proteins to mitigate the oxidative and inflammatory damage induced by DOX. These findings revealed that although SYA demonstrated a lower binding affinity than DOX, it formed stable interactions with all four targets, suggesting its potential to activate this cytoprotective signaling cascade. These results complemented the in vivo findings, supporting the mechanistic hypothesis that SYA confers hepatoprotection through modulation of the PI3K/Akt/Nrf-2/HO-1 axis. Thus, the docking studies bridged the gap between the data on molecular interactions and the observed physiological effects, offering a plausible mechanistic explanation for SYA’s antioxidant and protective actions. These findings suggested that DOX and SYA may exert their biological effects through multiple complementary mechanisms. The strong binding affinities observed, especially for DOX, along with the ADMET properties, indicated that these substances could have potential therapeutic applications and highlighted important considerations for drug development. The interaction patterns across different protein targets suggested potential poly-pharmacological effects in complex disease contexts where the modulation of multiple pathways is desired.

The B.W.C% in rats given DOX was significantly decreased. This could be attributed to the side effects of DOX that affect body weight and its toxic effects on liver tissues, which cause appetite loss, metabolism disturbances, disrupted protein turnover, and fatigue. Treatment with different doses of SYA resulted in a significant improvement in B.W.C% in a dose-dependent manner. These findings were consistent with previous reports on the effects of natural constituents on body weight alterations promoted by DOX chemotherapy [12,31,36,37]. Inflammatory reactions and compensatory mechanisms that support liver regeneration were observed because of DOX-induced liver injury, leading to an increase in liver organ weight. This was caused by the accumulation of components altering liver structures, which resulted in hepatocellular hypertrophy, vacuolation, bile duct hyperplasia, and necrosis [38,39].

The intraperitoneal injection of 4 mg/kg of DOX in male rats led to a significant enhancement of the serum activities of cytoplasmic, mitochondrial, and membrane-bound liver enzymes (ALT, AST, ALP, and GGT), indicating hepatic cell damage had occurred and led to leakage of the enzymes into the circulation and central vein congestion [40]. Conversely, the diminished protein levels were attributed to oxidative stress and inflammation triggered by DOX in liver tissues, which led to reduced liver function, hepatocellular damage, and hepatobiliary deterioration. Our findings were consistent with previous reports confirming the increase in these enzymes following DOX injection in rats [3,12,31,38,39,40]. Protein metabolism and turnover may change; even though the total protein content was reduced, the liver may undergo processes such as protein degradation more quickly due to oxidative protein damage. Damage to the liver may cause a drop in protein concentration, but the liver may still initiate processes such as enhanced glycogen storage or lipid accumulation to preserve its mass and function [2]. Treating DOX-injected rats with different concentrations of SYA led to significant mitigative effects on the elevated ALT, AST, ALP, and GGT serum levels, suggesting the potential therapeutic properties of SYA against DOX-triggered hepatotoxicity. Motamedi et al. (2024) highlighted the ameliorative effects of zingerone phenolic compounds on DOX-induced liver damage [41].

Oxidative stress induced by DOX led to the inhibition of hepatic antioxidant armory, causing the excessive production of free radicals and providing a cellular signal to disrupt oxidant/antioxidant hemostasis [42]. This oxidative stress in liver tissues can be mitigated by standby defense antioxidants boosted by natural compounds, which utilize toxic chemicals by inhibiting ROS triggering [43]. In this study, the elevated hepatic MDA levels and decreased hepatic levels of SODs, GSHs, GSTs, and GPXs in rats given DOX alone suggested that DOX had induced lipid peroxidation and oxidative stress in the rats’ hepatocytes. However, concomitant treatment with DOX and SYA, especially at the higher dosage, significantly restored the balance of oxidants and antioxidants in the rats’ livers. These findings confirmed the antioxidant power of SYA against oxidative damage in hepatic cells induced by DOX in rats, in accordance with previous studies [41,44,45,46]. Additionally, SYA alleviated oxidative damage more effectively than standard ascorbic acid by enhancing antioxidants in a rat model of hepatotoxicity [22]. Therefore, this investigation was extended to evaluate the limiting effects of SYA treatment on DOX-induced inflammation, which could be beneficial in alleviating DOX-induced hepatotoxicity [31].

Interleukins trigger the recruitment of inflammatory cells in liver tissues, promoting the production of other pro-inflammatory cytokines and inducing hepatocyte damage by stimulating hepatic stellate cells and diminishing liver function [47]. TNF-α induces a cascade of intracellular events that can result in cell death through increased ROS generation in liver tissues, further exacerbating oxidative stress and hepatic damage. The development of hepatocellular damage is influenced by NF-κB, a master regulator of inflammation and cell death [48]. The results reported that hepatic cytokines were significantly increased in the DOX-challenged group; however, by administering SYA, these biomarkers were decreased, with the most improvement recorded with the treatment of DOX and SYA at a dosage of 75 mg/kg. These mechanisms may contribute to SYA’s ability to attenuate the activation of inflammatory responses to DOX by preventing macrophages and other inflammatory cells from being activated, reducing the synthesis of cytokines and chemokines that sustain the inflammatory response in rat liver tissues. The anti-inflammatory effects of SYA treatment during liver injury have been reported in rats [20]. Consistent with our findings, previous studies have reported the reduction in inflammation upon treating DOX-administered rats with natural compounds [4,7,11,12,31,43,49].

Upstream of numerous protective pathways, the PI3K/Akt signaling pathway promotes cell survival [50]. The Nrf-2/HO-1 signaling route can be activated by the PI3K/Akt signaling pathway, which can also suppress cell death and promote cell proliferation [51]. An intriguing function of the transcription factor Nrf-2 is the promotion of antioxidant enzymes, such as SOD, HO-1, and GPX, to detect oxidative stress in liver cells [52]. It is unclear exactly what mechanism underlies SYA’s protective benefits. To explore the biochemical and molecular mechanisms of the protective effect of SYA on DOX-promoted hepatotoxicity, PI3K/Akt-mediated Nrf-2/HO-1 signaling pathway-related proteins, as well as their gene expression levels, were evaluated in relation to DOX and/or SYA administration in rats’ livers. DOX therapy has been shown to decrease the expression of Nrf-2 mRNA and proteins, which also decreases the expression of downstream antioxidant genes and proteins, resulting in liver damage [53]. In agreement with prior studies, the levels of these proteins were found to be significantly decreased in the DOX group, while SYA upregulated the PI3K, Akt, Nrf-2, and HO-1 expression levels with a more pronounced protective effect when the highest dosage of SYA (75 mg/kg) was given [3,53,54]. Zhang et al. (2024) reported that SYA treatment led to potential protective effects against nonalcoholic fatty liver in rats through its modulation of the Nrf-2/HO-1 signaling pathway [20]. Histopathological examination of liver tissues further supported the beneficial effects of SYA treatment in managing DOX-induced hepatotoxicity. The liver tissues of DOX-treated rats showed significant hepatic degeneration and congestion of blood vessels, which was in accordance with previous reports [3,12,31]. Notably, treatment with SYA, especially at the higher dosage, significantly ameliorated these histopathological changes, resulting in improvement in the liver tissue architecture. These histopathological investigations in this study supported other biochemical findings and confirmed the hepatoprotective effects of SYA against DOX-promoted hepatotoxicity. Several previous studies reported the mitigative effects of SYA on histopathological alterations induced in liver tissues of experimental animals [18,19,20,21,22]. The current study employed a male Sprague–Dawley rat model, which is a well-established preclinical model for DOX-induced hepatotoxicity. This model successfully replicates key pathological features of DOX-induced liver injury, including oxidative stress, inflammation, and hepatocellular damage. It has inherent limitations in representing the genetic heterogeneity observed in human patients with DILI. Human susceptibility to DILI can be influenced by polymorphisms in genes encoding cytochrome P_450_ enzymes, drug transporters, and immune-related pathways. These variations contribute to the idiosyncratic aspect of DILI, which is difficult to replicate in inbred rodent strains that lack such interindividual variability [55,56]. Therefore, while our rat model was valuable for studying the intrinsic (dose- and mechanism-related) aspects of DOX hepatotoxicity, it did not fully capture the genetic predisposition factors that may modulate DILI risk in the clinical setting. Future studies incorporating humanized mouse models, genetically diverse animal strains, or in vitro systems using patient-derived hepatocytes could help address this limitation and provide translational relevance.

## 4. Materials and Methods

### 4.1. Chemicals

Syringic acid (cat. no. S6881, ≥95% HPLC) and doxorubicin hydrochloride (cat. no. D5220, 98–102% HPLC) were purchased from Sigma-Aldrich in Oakville, ON, Canada.

### 4.2. In Silico Studies

To forecast the pharmacokinetic characteristics of DOX and SYA, including their absorption, distribution, metabolism, excretion, and toxicity (ADMET), the ADMETlab 2.0 web server was fed their SMILES codes [57]. The AlphaFold website was used to create models after the protein structures for PI3K (Uniprot ID: O70173), Akt (Uniprot ID: P47196), GSK-3β (Uniprot ID: P18266), Nrf-2 (Uniprot ID: O54968), and HO-1 (Uniprot ID: P06762) were retrieved from the UniProt database [58]. The CB-DOCK2 server was employed to predict the binding sites for these proteins [59]. AutoDock Tool 1.5.7 was used to further process the obtained protein structures. The ligand compounds’ active constituents were extracted from the PubChem database in their corresponding structure–data file (SDF) forms. The Avogadro 1.2.0 program was then used to decrease these ligands by utilizing the Conjugate Gradient algorithm and the Force Field (MMFF94) [60].

### 4.3. Rats and Experimental Design

Forty adult male Sprague–Dawley rats (weighing 150 to 170 g and 5 to 6 weeks of age) were obtained from Helwan University, Egypt. Our study was conducted in compliance with the Faculty of Science, Tanta University Animal Care Committee (IACUC-SCI-TU-0400-2024-12-24), Egypt. Forty male Sprague–Dawley rats were divided into five groups of eight rats each: Gp1 was a negative control group and Gps2–5 was administered with 4 mg/kg of DOX intraperitoneally (i.p.) once a week for a month [61]. This dose was selected based on prior rodent models that demonstrated consistent hepatotoxic effects (i.e., oxidative stress, enzyme elevation, histopathological damage). Although this dosage appears high in absolute terms, it is biologically relevant when scaled to human doses using body surface area conversion according to FDA guidelines [62]. The conversion factor for rats to humans is approximately 6.2. Therefore, a dosage of 16 mg/kg in rats corresponds to a human equivalent dose (HED) of approximately 2.58 mg/kg total for a 60 kg human. This is a dosage comparable to cumulative DOX exposures used in cancer patients that are associated with clinical DILI, particularly after multiple cycles or in susceptible individuals [54]. DOX-induced DILI in patients is most often observed at cumulative doses ≥400–550 mg/m^2^, where hepatic enzyme elevations and liver dysfunction are reported, especially in the context of combination chemotherapy. Our experimental dose thus provided a relevant preclinical approximation of the mechanistic hepatotoxicity observed in humans, especially the intrinsic, dose-dependent component. Gp2 was left as a positive control group. Gps3-5 were orally administered with SYA (25, 50, and 75 mg/kg/day, respectively) [9]. After a month, all rats were placed in a chamber containing isoflurane until they became unconscious, and then samples were collected for further analysis.

### 4.4. Biochemical Analysis

Serum aspartate aminotransferase (AST) (cat. no. AS106145), alanine aminotransferase (ALT) (cat. no. AL103145), alkaline phosphatase (ALP) (cat. no. AP1020), and gamma-glutamyl transferase (GGT) (cat. no. 246002), total proteins (cat. no. 310001), hepatic malondialdehyde (MDA) (cat. no. MD2529), superoxide dismutase (SOD) (cat. no. SD2521), catalase (CAT) (cat. no. CA2517), and reduced glutathione (GSH) (cat. no. GR2511) were assessed using bio-diagnostic kits from Cairo, Egypt. Liver homogenates were used to detect interleukin-6 (IL-6) (catalog no. E-HSEL-R0004), interleukin-1β (IL-1β) (cat. no. E-EL-R0012), tumor necrosis factor-α (TNF-α) (cat. no. RAB0479), and nuclear factor kappa-B (NF-κB) (cat. no. MBS453975) using their rat ELISA kits from My-BioSource Inc., (San Diego, CA, USA). Hepatic levels of rats’ p-PI3K (cat. no. MBS9518759), p-Akt (cat. no. MBS9518725), Nrf-2 (cat. no. MBS752046), and HO-1 (cat. no. MBS764989) were determined by using rat-specific ELISA kits. Protein concentrations were determined using the method of Lowry et al. (1951) [63].

### 4.5. Molecular Analysis

Using GAPDH as an internal reference, the mRNA expressions of the PI3K, Akt, Nrf2, and HO1 genes were assessed in the liver tissues. The NCBI Primer-Blast tool was used to create the primers (see Table 6). The relative expression of the target gene was calculated [64].

### 4.6. Histopathological Investigations

Liver tissues were fixed in 10% buffered formalin and then 5 μm sections were embedded in paraffin wax and washed in xylene. Hematoxylin and eosin (H&E) staining was applied. The sections were examined under an Olympus CX31 light microscope and captured on a digital camera (Olympus Camedia 5060, Tokyo, Japan) [65]. The percentage and severity of tissue damage were used to analyze hepatic injury. A scale of 0 to 4 was used, where 0 represents normal tissue, 1 represents less than 25% of damaged hepatic tissue, 2 represents 26–50% damage, 3 represents 51–75% hepatic damage, and 4 represents more than 75% damage [66].

### 4.7. Statistical Analysis

A mean ± standard error of the mean (SEM) was used to present the readouts. The Tukey test was conducted for multiple comparisons following one-way analysis of variance (ANOVA) or Kruskal–Wallis tests to determine significant differences. *p* ˂ 0.05 values were regarded as statistically significant. A sample size of eight rats per group, based on power analysis calculation, offers enough power (approximately 0.80) to detect medium-sized effects (Cohen’s f = 0.25), with α = 0.05.

## 5. Conclusions

This study demonstrated that treatment with SYA has potential alleviative effects on liver injuries caused by DOX injection. It combats oxidative stress, inflammation, and modulation of the PI3K/Akt-mediated Nrf-2/HO-1 signaling pathway. The combination of SYA and DOX could be a new and efficient form of chemotherapy that could be used against several types of cancer. However, further studies should investigate the efficacy of SYA, its underlying mechanisms, and its optimal therapeutic regimens.

## Figures and Tables

**Table 1:** The ADMET analysis of each syringic acid (SYA) and doxorubicin (DOX).

Name	Syringic Acid (SYA)	Doxorubicin (DOX)
LogS	−1.975	−2.69
LogD	3.978	0.689
LogP	1.212	1.76
Pgp-inh	0.002	0.024
Pgp-sub	0.003	0.999
HIA	0.028	0.751
F(20%)	0.011	0.024
F(30%)	0.067	0.168
Caco-2	−5.142	−6.006
MDCK	1.09 × 10^5^	6.24 × 10^6^
PPB	50.89%	91.29%
VDss	0.459	1.01
Fu	38.57%	12.38%
CYP1A2-inh	0.032	0.489
CYP1A2-sub	0.911	0.452
CYP2C19-inh	0.025	0.013
CYP2C19-sub	0.058	0.064
CYP2C9-inh	0.028	0.016
CYP2C9-sub	0.131	0.431
CYP2D6-inh	0.012	0.005
CYP2D6-sub	0.141	0.168
CYP3A4-inh	0.016	0.103
CL	7.208	9.566
T12	0.946	0.847
hERG	0.034	0.019
H-HT	0.154	0.257
DILI	0.795	0.964
Ames	0.009	0.811
ROA	0.011	0.022
FDAMDD	0.023	0.453
SkinSen	0.129	0.418
Carcinogenicity	0.034	0.776
EC	0.292	0.003
EI	0.977	0.073
Respiratory	0.044	0.92
BCF	0.401	0.451
IGC_50_	2.487	3.709
LC_50_	2.55	3.784
LC_50_DM	3.357	5.493
NR-AR	0.012	0.017
NR-AR-LBD	0.046	0.964
NR-AhR	0.196	0.906
NR-Aromatase	0.021	0.834
NR-ER	0.112	0.619
NR-ER-LBD	0.007	0.536
NR-PPAR-gamma	0.235	0.165
SR-ARE	0.061	0.813
SR-ATAD5	0.191	0.549
SR-HSE	0.139	0.01
SR-MMP	0.043	0.959
SR-p53	0.053	0.988
MW	198.05	543.17
Vol	189.069	516.727
Dense	1.048	1.051
nHA	5	12
nHD	2	9
TPSA	75.99	212.39
nRot	3	5
nRing	1	5
MaxRing	6	18
nHet	5	12
nRig	7	28
Flex	0.429	0.179
nStereo	0	5
NonBiodegradable	0	3
SureChEMBL	0	1
Skin_Sensitization	4	8
Toxicophores	1	3
Genotoxic_Carcinogenicity_Mutagenicity	0	4
QED	0.76	0.147
Synth	1.726	5.015
Fsp3	0.222	0.37
MCE-18	8	118.243
Natural Product-likeness	0.544	1.37
Lipinski	Accepted	Rejected
Pfizer	Accepted	Accepted
GlaxoSmithKline	Accepted	Rejected

HIA: human intestinal absorption; F (%): oral bioavailability; PPB: plasma protein binding; VDss: volume of distribution; Fu: fraction unbound; CYP1A1: cytochrome P450 family 1 subfamily A; CL: clearance; T1/2: half life time; ROA: route of administration; EI: enzyme induction; BCF: bioconcentration factor; LC_50_: median lethal concentration; ER: extended-release; SR: sustained-release; HSE: health, safety, and environment; MMP: matrix metalloproteinase inhibitor; MW: molecular weight; nHA: number of hydrogen bond acceptors; nHD: number of hydrogen bond donor; TPSA: topological polar surface area; nROT: number of rotatable bonds; nHet: number of heteroatoms; MaxRing: number of atoms in the biggest ring; nRing: number of rings; QED: quantitative estimate of drug-likeness.

**Table 2:** The ∆G and molecular docking binding affinity (kcal/mol) of DOX and SYA.

Compound/Protein	PI3K	Akt	Nrf-2	HO-1
DOX	−6.5	−7.9	−6.9	−7.7
SYA	−4.4	−5.6	−4.5	−5.4

DOX: Doxorubicin; SYA: Syringic acid; PI3K: Phosphatidylinositol 3-kinase; Akt: Protein kinase B; Nrf-2: Nuclear factor erythroid 2–related factor 2; HO-1: Hem-oxygenase 1.

**Table 3:** The initial, final body weights, the percentages of the body weight change, and relative liver weight after different treatments.

Groups	Control	DOX	DOX/SYA (25 mg/kg)	DOX/SYA (50 mg/kg)	DOX/SYA (75 mg/kg)
I.B.W (g)	155.21 ± 6.54 ^a^	162.74 ± 5.83 ^a^	165.42 ± 4.87 ^a^	157.62 ± 5.81 ^a^	160.78 ± 6.23 ^a^
F.B.W (g)	215.23 ± 7.69 ^a^	185.44 ± 8.16 ^c^	195.12 ± 6.15 ^a,c^	196.47 ± 5.79 ^a,c^	204.77 ± 7.12 ^a^
B.W.C (%)	38.71% ^a^	13.94% ^e^	17.95% ^e,c^	24.64% ^e^	27.40% ^a,e^
R.L.W (%)	4.61 ± 0.25 ^e^	5.07 ± 0.31 ^e^	4.87 ± 0.29 ^e^	4.71 ± 0.33 ^e^	4.70 ± 0.27 ^e^

The values are expressed as mean ± SEM (*n* = 8). DOX: Doxorubicin; SYA: Syringic acid; I.B.W: Initial body weight; F.B.W: Final body weight; B.W.C: Body weight change; R.L.W: Relative liver weight. Means with different lowercase letters indicate statistically significant differences between groups (*p* < 0.05), as determined by Tukey’s HSD post hoc test following one-way ANOVA. Groups sharing the same letter are not significantly different.

**Table 4:** Serum alanine transaminase (ALT), aspartate transaminase (AST), alkaline phosphatase (ALP), Gamma-glutamyl transferase (GGT), and total protein (TP) in different groups.

Groups	AST (U/L)	ALT (U/L)	ALP (U/L)	GGT (U/L)	TP (mg/dL)
Control	48.38 ± 3.16 ^c^	34.67 ± 2.25 ^a^	192.83 ± 4.87 ^a^	7.64 ± 0.79 ^b^	8.19 ± 0.65 ^c^
DOX	114.85 ± 4.87 ^e^	89.64 ± 3.69 ^c^	325.68 ± 6.93 ^b^	19.14 ± 1.95 ^a^	3.95 ± 0.29 ^b^
DOX/SYA (25 mg/kg)	81.65 ± 3.78 ^a^	68.82 ± 2.97 ^e^	274.73 ± 6.88 ^c^	11.05 ± 1.48 ^c^	5.54 ± 0.44 ^a^
DOX/SYA (50 mg/kg)	69.74 ± 3.56 ^a^	60.84 ± 3.12 ^e^	242.61 ± 4.79 ^c^	9.23 ± 1.17 ^b^	6.21 ± 0.64 ^a^
DOX/SYA (75 mg/kg)	60.93 ± 3.17 ^c,d^	53.69 ± 2.95 ^e,d^	239.33 ± 5.94 ^c^	10.46 ± 1.08 ^b,c^	5.93 ± 0.59 ^a^

The values are expressed as mean ± SEM (*n* = 8). DOX: Doxorubicin; SYA: Syringic acid. Means with different lowercase letters indicate statistically significant differences between groups (*p* < 0.05), as determined by Tukey’s HSD post hoc test following one-way ANOVA. Groups sharing the same letter are not significantly different.

**Table 5:** Hepatic malondialdehyde (MDA), superoxide dismutase (SOD), glutathione S-transferase (GST), glutathione peroxidase (GPX), and reduced glutathione (GSH) levels in the different groups.

Groups	MDA (nmol/mg Protein)	SOD (U/mg Protein)	GST (U/mg Protein)	GPX (U/mg Protein)	GSH (nmol/mg Protein)
Control	2.16 ± 0.18 ^a^	12.35 ± 0.95 ^c^	36.45 ± 1.48 ^c^	73.69 ± 3.28 ^b^	24.87 ± 1.54 ^c^
DOX	6.85 ± 0.57 ^b^	5.79 ± 0.48 ^a^	17.29 ± 1.12 ^d^	41.58 ± 2.97 ^a^	10.79 ± 1.15 ^b^
DOX/SYA (25 mg/kg)	5.57 ± 0.39 ^b^	8.13 ± 0.66 ^b^	25.37 ± 1.74 ^a^	52.74 ± 2.65 ^c^	16.15 ± 1.64 ^a^
DOX/SYA (50 mg/kg)	4.41 ± 0.51 ^b, c^	9.37 ± 0.58 ^b^	29.82 ± 2.01 ^a^	64.18 ± 3.14 ^b,c^	19.07 ± 1.90 ^a^
DOX/SYA (75 mg/kg)	3.29 ± 0.33 ^a,c^	10.95 ± 1.06 ^b,c^	33.44 ± 1.95 ^c^	67.95 ± 2.48 ^b,c^	19.93 ± 1.84 ^a^

The values are expressed as mean ± SEM (*n* = 8). DOX: Doxorubicin; SYA: Syringic acid. Means with different lowercase letters indicate statistically significant differences between groups (*p* < 0.05), as determined by Tukey’s HSD post hoc test following one-way ANOVA. Groups sharing the same letter are not significantly different.

**Table 6:** Forward and reverse primer sequences for RT-PCR.

Gene	Accession Number	Forward Sequence (5′-3′)	Reverse Sequence (5′-3′)
*PI3K*	NM_053481.2	CGAGAGTACGCTGTAGGCTG	AGAAACTGGCCAATCCTCCG
*Akt1*	NM_033230.3	GAAGGAGAAGGCCACAGGTC	TTCTGCAGGACACGGTTCTC
*Nrf2*	NM_031789.3	CACATCCAGACAGACACCAGT	CTACAAATGGGAATGTCTCTGC
*HO1*	NM_012580.2	ACAGGGTGACAGAAGAGGCTAA	CTGTGAGGGACTCTGGTCTTTG
*GAPDH*	NM_017008.4	CCGCATCTTCTTGTGCAGTG	GAGAAGGCAGCCCTGGTAAC

*PI3K*: phosphoinositide 3-kinase, *Akt*: protein kinase B, *Nrf-2*: nuclear factor erythroid 2-related factor 2, *HO-1*: heme oxygenase 1, *GAPDH*: glyceraldehyde-3-phosphate dehydrogenase.

## Data Availability

The data presented in this study are available on request from the corresponding author. The data are not publicly available due to ethical restrictions; our research can still be used in ongoing studies or future research, and sharing it prematurely could jeopardize the intellectual property or future publications.
